# Who deserves credit, who receives credit? A cross-sectional survey on the handling of co-authorship in medical dissertations in Germany

**DOI:** 10.1186/s41073-026-00219-w

**Published:** 2026-05-22

**Authors:** Laura Klempp, Nadin Zerin Tanriverdi, Adrian Loerbroks, Niklas Juth, Nils Hansson

**Affiliations:** 1 Department of the History, Philosophy and Ethics of Medicine, Centre for Health and Society, Medical Faculty, Heinrich-Heine-University, Moorenstraße 5, Duesseldorf, 40225 Germany; 2 Institute of Occupational, Social and Environmental Medicine, Centre for Health and Society, Medical Faculty, Heinrich-Heine-University Duesseldorf, Duesseldorf, Germany; 3 Department of Public Health and Caring Sciences, Centre for Research Ethics and Bioethics, Uppsala University, Uppsala, Sweden

**Keywords:** Authorship, Research ethics, Research ethical guidelines, Mentorship, Gender discrimination

## Abstract

**Background:**

This study investigates the awareness and application of the internationally recognized Vancouver guidelines on co-authorship among doctoral students in medicine across North Rhine-Westphalia (NRW), Germany, the federal state with the highest number of medical faculties (nine) nationwide. Although authorship practices have been widely discussed in medical research, empirical data on doctoral students’ awareness of and adherence to these guidelines remain limited, particularly in Germany.

**Methods:**

We carried out a cross-sectional online survey among 147 former doctoral students who completed their dissertations at a medical faculty during the last two years. Participants were recruited via mailing lists, social media, and doctoral graduation ceremonies between December 2024 and June 2025. Descriptive statistics were used to summarize the data. To examine further relationships, correlational analyses were conducted using chi-square tests.

**Results:**

While 84% (*n* = 123/147) of the participants considered the Vancouver authorship guidelines important, 61% (*n* = 90/147) reported that at least one criterion of the Vancouver guidelines had not been fulfilled in their work. Furthermore, 56% (*n* = 82/147) reported never having received any information about the guidelines. Among doctoral students supervised by male supervisors (*n* = 103), female doctoral students had lower odds of receiving information about authorship guidelines (Odds Ratio [OR] = 0.37, 95% CI 0.17—0.83) and of reporting a clear policy regarding them (OR = 0.37, 95% CI 0.16—0.81) compared with male doctoral students.

**Conclusion:**

Overall, our findings indicate that, among the surveyed cohort of former medical doctoral students in Germany, self-reported adherence to authorship guidelines was limited and observed gender-related differences. Variations in the perceived dissemination and implementation of ethical authorship practices were associated with the supervisory gender constellation. These results underscore the need for more robust institutional frameworks, systematic education on authorship ethics, and clearer accountability mechanisms in the supervision of doctoral research.

**Supplementary Information:**

The online version contains supplementary material available at 10.1186/s41073-026-00219-w.

## Background

The tongue-in-cheek aphorism *“publish or perish”* underscores the crucial role of scientific publications and citations in shaping academic visibility and career progression [1, 2]. The reasons for the growing number of co-authors per paper in the life sciences are multifaceted [3, 4]. Scholars assumed that this trend is driven by increasing interdisciplinary collaboration and the fact that multi-authored papers are cited more frequently and are more likely to appear in high-impact journals [3, 5]. However, while longer author lists may reflect the collaborative nature of modern research, they also raise concerns about gift or honorary authorship, where individuals are listed as co-authors despite having made little or no meaningful contribution to the work [6]. Such practices may be driven by systemic factors including academic hierarchies, pressures to publish, and reward structures that value publication quantity over ethical authorship [2]. Previous studies have shown that senior academics, particularly full professors and department heads, often benefit disproportionately from this practice [7–9]. These developments have reignited longstanding debates around the meaning of authorship and the author ordering. In medicine, author order typically follows a convention in which the first author usually makes the largest contributions, while the last (senior) author, typically the group leader, assumes overall responsibility [10, 11]. This issue is particularly relevant for doctoral candidates, for whom the dissertation often constitutes an initial entry point into scientific research and plays a central role in academic socialization. Authorship embodies both scientific responsibility and scholarly recognition, frequently serving as a gateway to academic research and a key stepping stone in early career development.

To promote ethical publishing practices, the International Committee of Medical Journal Editors (ICMJE) has established clear guidelines outlining the criteria for authorship, the so-called Vancouver guidelines [12]. According to the ICMJE, authorship requires fulfilling four criteria: making a substantial intellectual contribution to the study’s conception, design, or analysis; contributing to drafting or critically revising the manuscript; approving the final version; and taking accountability for the integrity of the entire work. [12] Failure to meet these conditions constitutes a violation of ethical authorship standards. However, one problem that becomes evident is that the mere existence of the guidelines does not guarantee their implementation [7]. These guidelines must first be known, understood, and recognized as binding by all participants in the research process [7].

During the last three decades, studies have examined whether the principles of 'good scientific practice', including ethical authorship, are known and adhered to by medical researchers, consistently finding low awareness and limited compliance. As early as in 1997, a UK-based survey revealed minimal familiarity with ICMJE authorship criteria among medical students [13]. In Jordan, roughly only 22% of medical students were aware of the guidelines [14], while in Pakistan the figure was around 10% [15]. A 2018 study from Sweden reported that 53% of medical dissertations did not fully comply with the ICMJE guidelines [16]. A follow up study in Scandinavia presented similar results (46%) [17].

It is not only the mere non-compliance with the guidelines that is problematic, but also their relevance in relation to gender-specific patterns. Gender-specific inequalities in supervision and collaboration contribute to unequal opportunities in the publication of work [18, 19], with male doctoral students tending to receive more recognition [20] and female doctoral students publishing less under male supervisors [21]. Even though the number of female authors in medicine continues to rise, studies show inequalities in the distribution of authorship here as well [18, 22].

In this study, we surveyed scholars in Germany, who completed their dissertations at a medical faculty during the last two years.

Medical dissertations in Germany differ from doctoral research in other disciplines and countries in some respects. First, the pursuit of a doctoral degree in Germany is substantially more common in medicine than in most other fields. Although medical students account for only 7.22% of all students in Germany [23], they represent approximately 28% of all doctoral students [24]. Second, many medical doctoral students begin their dissertations during undergraduate studies. These projects are typically embedded within research groups, and German medical faculties tend to be more hierarchical than those in disciplines such as the arts and humanities.

Our aim was to assess their knowledge of and experience with ethical authorship guidelines. By doing so, we sought to identify current trends and highlight potential challenges faced by doctoral students in navigating authorship research ethics within the framework of academic medicine.

## Methods

### Recruitment

A cross-sectional online survey was conducted between December 6, 2024, and June 27, 2025 in accordance with the STROBE guidelines (see additional file 1 for the completed checklist). The study was reviewed and approved by the Ethics Committee of the Medical Faculty of Heinrich Heine University Düsseldorf, Study number 2024—3071. Doctoral students were eligible if they had received a Dr. med. (physicians), Dr. med. dent. (dentists), Dr. PH (public health), or a PhD degree in 2024 or 2025 from one of the nine medical faculties in NRW, Germany, through cumulative dissertations (i.e., based on published journal articles). Those with monograph-based dissertations were thus excluded, since those have only one author. It is important to note that the specific requirements for the number of publications for a cumulative dissertation vary depending on the doctoral degree pursued. In Duesseldorf for example, for the degrees Dr. med., Dr. med. dent., and Dr. PH, at least one original research article with first authorship is required. In contrast, for the PhD, a minimum of three original research articles is required, of which at least two with first authorship.

**Fig. 1:**
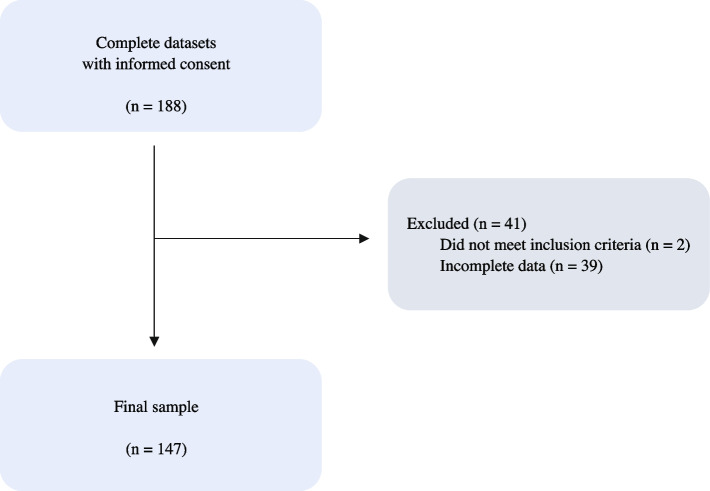
Flow diagram. Flow diagram illustrating the progression from all conducted interviews to the final analytical sample

We contacted the doctoral offices of all nine medical faculties in North Rhine-Westphalia (NRW), Germany, the country’s most populous federal state and the one with the highest number of medical faculties (viz. nine) nationwide, requesting to distribute the questionnaire. Overall, six universities replied. Doctoral offices at four medical faculties (i.e., Düsseldorf, Duisburg/Essen, Bonn and Münster) supported our study and shared the link to the questionnaire via mailing lists. Additionally, the first author (LK) recruited participants in person at doctoral graduation ceremonies in Bonn, Münster and Düsseldorf. The survey was also shared via social media and messenger groups in Düsseldorf, Aachen, Cologne and Bochum. In Aachen, participants were recruited through doctoral representatives at the institutes, who distributed the survey. In Cologne, recruitment took place via institute websites, while in Bochum, the study was promoted through the May newsletter and on social media. As the recruiting link was publicly shared through multiple channels, it was not possible to determine precisely which recruitment strategy generated individual responses. The flow of participants is shown in Fig. 1.

### Survey design and distribution

The online survey was created using SoSci Survey [25] and was accessible via a link or QR code. Completion of the survey took approximately 10 min, and all data were collected anonymously. The questionnaire comprised 15 items and two free-text fields. It was developed based on an instrument used in a Swedish study [16] and expanded to include additional questions. The survey began with the collection of sociodemographic and academic characteristics, including gender, authorship position, type of doctorate, university, year of graduation, and supervisor’s gender. This was followed by information on the Vancouver guidelines. The questionnaire then assessed whether the respondents had received information on these guidelines, whether clear departmental policies existed, and how reasonable and important they considered them. It further asked whether individuals not meeting ICMJE criteria were listed as authors and whether authorship order reflected relative contributions and should be adhered to. The translated questionnaire is provided in the appendix.

The questionnaire was pretested with six individuals of both genders, including doctoral students, resulting to minor wording adjustments. In total, 188 doctoral students completed the survey and provided informed consent. After exclusion of those with incomplete data (*n* = 39), data from one non-NRW doctoral student, and one with monographic dissertation, data from 147 respondents were available for the statistical analyses.

### Data analysis

Data were analyzed using SPSS version 31.0. After cleaning and filtering incomplete responses, prevalences were calculated. As part of the descriptive analysis, we generated two new variables: “at least one criterion was not met” and “at least two criteria were not met”. For these variables, responses of “unsure” and “no” were both coded as “no”, so that only a definitive “yes” was considered as an indication of a violation. In addition, we created a separate variable capturing violations of authorship order. For this variable, “no” and “unsure” responses were likewise combined into a single category (“no”), with only a clear “yes” response indicating an authorship order violation. Prior to this, a new variable was created for the outcome “clear policy on co-authorship”. Response options “yes, consistently” and “yes, partially” were combined into a single “yes” category, whereas “no” and “I don’t know/unsure” were combined into a single “no” category.

All variables included in the analyses were categorial and analyzed as frequencies (counts), no mean values were calculated. Accordingly, chi-square tests were used to examine associations between indicators of awareness and implementation of authorship guidelines (i.e., information received, departmental policy, adherence to authorship order) questionnaire responses and authorship positions, gender constellations and dissertation fields. The level of statistical significance was set at alpha = 0.05. Assumptions for chi-square testing were met. In addition, odds ratios (ORs) with 95% confidence intervals were computed from 2 × 2 contingency tables to provide effect size estimates and improve the interpretability of the chi-square results. For those analyses, some variables were grouped:

i) Authorship roles were categorized into “first author” and a combined “other author positions” category, which included “last author”, “corresponding author”, and “other”. This grouping was applied to examine, through comparative analysis, whether the position of first authorship, due to its distinct advantages, differs from other authorship positions.

ii) Dissertation fields were grouped into “Basic research”, “Clinical”, and “Mixed”. This grouping was based on the 2018 Swedish study by Helgesson et al. [16], to enable comparisons between the respective groups in terms of the dissertation fields. Twenty-eight respondents stated that their thesis belonged to other areas and were therefore excluded from this specific comparison. The resulting sample (*n* = 119) was used as the reference population (100%) for this analysis. For all other analyses, the full sample (*n* = 147) was retained.

iii) For the analysis of supervisory gender constellations, we first created gender variables for both doctoral students and supervisors. We included only respondents who identified as either male or female, as no responses indicated a non-binary gender. Based on these variables, we created combined variables representing candidate-supervisor gender constellations (e.g., “female-female”, “female-male”, “male–female”, “male-male”). To examine differences across doctoral student – supervisor gender constellations, chi-square tests were first conducted using the full four-category variable (female-female, female-male, male–female, male-male).

As the overall chi-square test does not indicate which specific groups may differ, additional pairwise comparisons were conducted to further explore subgroup differences. Based on prior literature [20, 21] and theoretical considerations, a focused comparison between female and male doctoral students with male supervisors was performed. Separate analyses were conducted for each outcome variable (receiving information on guidelines, clear policy on co-authorship and first authorship), reflecting distinct research questions. Given the focused, literature-informed nature of the analysis and the conceptual independence of the outcome variables, no adjustment for multiple testing was applied. Due to the insufficient number of open-ended responses, these were not further analyzed or evaluated and were therefore not included in the results.

## Results

### Sample characteristics

The distribution of measured characteristics of our sample is largely in keeping with the distribution in the reference population, that is, more than half of the participants were female (55%), most reported to have been first authors (74%) and pursuit of the title “Dr. med.” was most common (78%). The majority came from Heinrich Heine University Düsseldorf (40%), University of Münster (33%,) and University of Duisburg-Essen (10%,), with the remaining participants distributed across other institutions. Clinical research was the most common dissertation field reported by participants (42%), followed by basic research (27%) Table 1.

**Table 1:** Sample characteristics (*n* = 147)

Characteristics	n (%)
**Sex, n (%)**	
Female	81 (55%)
Male	66 (45%)
Divers	0
**Author position, n (%)**	
First author	109 (74%)
Corresponding author	21 (14%)
Last author	1 (1%)
Other	16 (11%)
**Type of promotion, n (%)**	
Dr. med.	115 (78%)
Dr. med. dent.	14 (10%)
PHD	12 (8%)
Dr. PH	6 (4%)
**Field of dissertation, n (%)**	
Clinical research	61 (42%)
Basic research	39 (27%)
Mixed (basic and clinical/applied research)	19 (13%)
Other applied research	17 (12%)
Other	11 (8%)
**Place of defense, n (%)**	
Dusseldorf	58 (40%)
Munster	49 (33%)
Duisburg-Essen	14 (10%)
Aachen	9 (6%)
Cologne	9 (6%)
Bonn	8 (5%)

Dr. med. (physicians), Dr. med. dent. (dentists), and Dr. PH (public health) require at least one original publication with first authorship; the PhD requires a minimum of three, including at least two with first authorship

### Views on authorship (frequencies)

More than half of the participants (56%, *n* = 82) indicated that they had not received any information about the Vancouver guidelines or other ethical guidelines on co-authorship as doctoral students. However, 53% (*n* = 78) reported that guidelines and strategies for co-authorship were applied in their department to varying degrees. All respondents agreed that the guidelines were reasonable.

As much as 61% (*n* = 90) reported that at least one authorship criterion was not met in the dissertation papers, and 46% (*n* = 68), reported at least two unmet criteria.

About one third (34%, *n* = 50) reported that they felt that the order of authors did not correspond to their respective relative contributions. In this context, 88% (*n* = 129) of participants stated that they considered it important for the order of authors to reflect the relative contributions of the authors to each other (43% answered “very important”, 45% answered “quite important”). Table 2.

**Table 2:** Views on authorship (*n* = 147)

Views on authorship	Answer	n (%)
Information about the Vancouver guidelines or other ethical guidelines on co-authorship received	Yes No	65 (44%) 82 (56%)
Clear guidelines or strategies for co-authorship available at department of doctorate	Yes, consistently	24 (16%)
	Yes, partially	54 (37%)
	No	44 (30%)
	I don’t know/unsure	25 (17%)
Consideration of the Vancouver guidelines for authorship as reasonable	Very reasonable	53 (36%)
	Quite reasonable	80 (54%)
	Partially reasonable	14 (10%)
	Not particularly reasonable	0
	Not reasonable at all	0
Inclusion as co-author on dissertation papers without significant contributions:		
a) Authorship without contribution to work design, data analysis, or interpretation	Yes	75 (51%)
	No	64 (44%)
	I don’t know/Unsure	8 (5%)
b) Authorship without writing or critical revision of the work		
	Yes	79 (54%)
	No	62 (42%)
	I don’t know/Unsure	6 (4%)
c) Authorship without final approval for publication	Yes	27 (18%)
	No	107 (73%)
	I don’t know/Unsure	13 (9%)
Importance of adherence to Vancouver guidelines for co-authorship	Very important	40 (27%)
	Quite important	83 (57%)
	Partially important	23 (16%)
	Not particularly important	1 (1%)
	Not important at all	0
Incorrect author order relative to contributions	Yes	50 (34%)
	No	84 (57%)
	I don’t know/Unsure	13 (9%)
Importance of author order reflecting relative contributions	Very important	63 (43%)
	Quite important	66 (45%)
	Not particularly important	16 (11%)
	Not important at all	2 (1%)

### First author vs. other author positions

Doctoral students with first authorship more frequently reported having received information about authorship guidelines compared to doctoral students in other authorship positions (51% (56/109) vs. 24% (9/38); OR = 3.41, 95% CI 1.47—7.86, *p* = 0.003).

Furthermore, first authors had lower odds of reporting violations of authorship order compared to doctoral students with other authorship position (25% (27/109) vs. 61% (23/38); OR = 0.22, 95% CI 0.10—0.50, *p* < 0.001). Additionally, doctoral students who were first authors more frequently reported that their department had clear co-authorship policies compared to students with other author positions (61% (66/109) vs. 32% (12/38); OR = 3.33, 95% CI 1.52—7.29, *p* = 0.002).

### Gender differences

The overall chi-square tests across all four doctoral student-supervisor gender constellations were not statistically significant (χ^2^ (3) = 7.19, *p* = 0.066, V = 0.22; χ^2^ (3) = 6.26, *p* = 0.099, V = 0.21; χ^2^ (3) = 5.82, *p* = 0.120, V = 0.19).

Given the exploratory nature of the analyses, post-hoc comparisons were conducted within the male-supervisor subgroup (*n* = 103). Within this subgroup, female doctoral students were less likely than male doctoral students to have received information on authorship guidelines (32% vs. 56%; OR = 0.37, 95% CI 0.17—0.83, *p* = 0.014) and to report a clear application of these guidelines (42% vs. 66%; OR = 0.37, 95% CI 0.16—0.81, *p* = 0.013). In both cases, male doctoral students more frequently reported being informed about authorship guidelines and having a clear policy regarding them.

A difference in first authorship was also observed, with first authorship more frequently reported among male doctoral students (84% vs. 64%), corresponding to lower odds among female doctoral students (OR = 0.34, 95% CI 0.13–0.87, *p* = 0.022). No statistically significant main effects were observed across other supervisor gender constellations. Table 3.

**Table 3:** Differences across doctoral student-supervisor gender pairings (*n* = 147)

Items n (%)	F. student + F. supervisor	F. student + M. supervisor	M. student + F. supervisor	M. student + M. supervisor	p
Received Information about ethical guidelines on authorship					.066
Yes	11 (8%)	17 (12%)	9 (6%)	28 (19%)	
No	17 (12%)	36 (25%)	7 (5%)	22 (15%)	
Clear policy on co-authorship					.099
Yes	15 (10%)	22 (15%)	8 (5%)	33 (22%)	
No	13 (9%)	31 (21%)	8 (5%)	17 (12%)	
Authorship position					.120
First position	22 (15%)	34 (23%)	11 (8%)	42 (29%)	
Other positions	6 (4%)	19 (13%)	5 (3%)	8 (5%)	

## Discussion

This study provides insight into the application of authorship guidelines as perceived and experienced by recent doctoral graduates in various medical schools in Germany. The findings suggest considerable gaps in the application of established authorship standards, especially when compared to a similar Swedish study conducted in 2018 by Helgesson et al. (*n* = 285) [16]. Both studies used comparable survey instruments to assess ICMJE authorship knowledge and targeted recent medical doctoral graduates. Although the 2018 Swedish study [16] benefited from centralized registries and higher response rates, whiles ours relied on open recruitment from NRW faculties, the alignment of key findings supports cross-national comparability. Violations of ICMJE criteria for authorship were more frequent in the German sample (*n* = 147): the first criterion, which includes conception, design, analysis and interpretation of data for the work, was violated according to 51% of the participants (Sweden: 39%) [16]. The second criterion, which includes drafting or reviewing the work, was violated in 54% (Sweden: 40%), and the third criterion, namely the final approval, was violated in 18% (Sweden: 14%) [16]. Authorship order was seen as inconsistent with actual contributions by 34% in Germany, compared to 28% in Sweden [16]. The Swedish sample also showed better dissemination of authorship guidelines [16]. While 80% of Swedish respondents had received information on authorship [16], only 44% in Germany had. Conversely, 56% of German participants reported having not received such information, compared to 15% in Sweden [16]. Departmental policies on authorship were reported similarly unclear in both countries. Attitudes towards the importance of adhering to the Vancouver guidelines also differed: 70% of Swedish respondents considered them very important [16], versus only 27% in Germany. However, 57% of German respondents saw them as “quite important”, compared to 27% in Sweden [16]. Unlike the Swedish study [16], the German data revealed few statistically significant differences by dissertation type (basic science, clinical, mixed). The only notable finding was a higher violation rate of the first criterion in clinical dissertations. Otherwise, differences across dissertation types were minimal.

The core findings align closely with a 2023 Scandinavian study on doctoral students in Sweden, Norway and Denmark [17]. As in our study, both the Swedish [16] and Scandinavian [17] research teams found a discrepancy between non-compliance with authorship criteria and the extent to which the guidelines were perceived as reasonable. Similar results have been reported in other countries: many doctoral students report being unfamiliar with the guidelines, and compliance is often lacking [14, 15]. Dissertation and publication cultures vary internationally, with different requirements for medical degrees. Nonetheless, national authorship criteria usually align with the ICMJE guidelines.

A striking result of our study is that 61% of the respondents reported at least one, and 46% at least two violations of authorship criteria in their cumulative dissertation. Despite the existence of ICMJE guidelines, adherence appears low, likely because of a lack of formal instruction: fewer than half reported receiving information about co-authorship guidelines. This lack of formal instruction may explain the high frequency of violations and the relatively low prioritization of strict adherence to authorship criteria. Without adequate guidance, students cannot be expected to follow guidelines they were never taught. This highlights the urgent need for better education and institutional enforcement of good scientific practice. However, even with awareness of guidelines, students often lack the power to apply them. Research suggests that hierarchical structures and power imbalances between supervisors and students may prevent fair authorship decisions [7].

These issues can have long-term effects on academic integrity. If students perceive authorship processes as unfair, they may be discouraged from pursuing research or perpetuate questionable practices. Compared to international benchmarks, our findings highlight the need for stronger institutional frameworks, consistent education on authorship ethics, and clearer accountability in supervising doctoral research.

Our findings describe gender-related differences in doctoral supervision; however, these observations are correlational and thus do not imply causal relationships. For example, the gender of supervisors is associated with differing reported experiences among male and female doctoral students. Male doctoral students more frequently report receiving authorship guidance in same-gender supervisory pairs, while female doctoral students supervised by men report receiving less information on authorship guidelines and exhibit lower odds of first authorship within the respective subgroup analyses. These observed associations may be influenced by unobserved or uncontrolled factors, including but not limited to disciplinary composition, variation in publication practices across fields, cohort effects, or differences in the perception and reporting of authorship related guidance. Accordingly, the findings should be interpreted as descriptive associations rather than explanatory mechanisms. Still, these patterns are consistent with prior studies describing a preference for same-gender collaborations in medical authorship, particularly among men [18]. For instance, male-only teams are significantly more common than female-only or male-led mixed-gender teams [18]. Similarly, Feldon et al. report that male doctoral students were 15% more likely to receive authorship credit than females [20], and Pezzoni et al. observe that female students published 8.5% fewer articles under male, but not female, supervisors [21]. Taken together, these studies suggest recurring patterns of gendered collaboration structures. Given that the large proportion of senior authors and supervisors in medical science are male [19, 20, 26], and that same-gender collaboration is commonly observed [18], these combined patterns may be consistent with previously described structural gender inequalities in academic publishing, without establishing direct causal mechanisms. Although female authorship has increased overall, growth remains slower among senior female authors [18, 22] which may reflect persistent structural barriers in academic progression. Taken together, these findings highlight patterns of association that may be relevant for understanding gender differences in early academic careers, particularly in male-dominated fields. Based on the observed correlations, we suggest strengthening authorship education, improving clarity and transparency through training and mentoring, and providing targeted support for female researchers, in line with recent work highlighting the need for clearer attribution of contributions and greater responsibility in authorship practices [27]. Further research is required to clarify underlying mechanisms.

## Limitations

This study has several limitations. Data were collected from only six of nine medical faculties in NRW, with relatively low response rates. Recruitment was hindered by differing program structures and limited access to the group of interests. A selection bias is possible, as those with publication-related concerns may have been more likely to respond, potentially overstating the issue. The exclusive focus on former doctoral students limits objectivity, as their responses reflect subjective perceptions. Supervisors’ perspectives were not included. Nonetheless, these perceptions highlight the need for better communication and education on ethical authorship. Given the exploratory nature of the study and limited prior evidence on gender and authorship among medical doctoral students in Germany, no adjustment for multiple testing (e.g., Bonferroni) was applied. Findings should be interpreted as hypothesis-generating for future research.

## Conclusion

Among recent medical doctoral graduates in Germany, adherence to ICMJE authorship guidelines appears to be limited. 61% of respondents reported at least one violation in the context of cumulative dissertations, most frequently related to criteria concerning substantial contributions, manuscript drafting, and final approval. In comparison with a similar study conducted in Sweden [16], German respondents demonstrated lower awareness of the guidelines (44% vs. 80% reporting familiarity) and attributed less importance to them, despite broadly comparable methodological approaches. These findings are consistent with evidence from Scandinavian [17] and other international studies, suggesting a more widespread deficit in formal education on authorship standards [13–15]. Gender-related disparities further complicate authorship practices. Female doctoral students supervised by male principal investigators reported receiving less information and were less likely to attain first authorship, in line with prior research indicating a tendency toward homophilic collaboration patterns [18]. To address these challenges, we argue that medical faculties should implement mandatory training on authorship and publication ethics at an early stage of doctoral education, establish transparent and enforceable departmental policies, and strengthen accountability in supervision. In addition, targeted measures to support female doctoral researchers may be needed to mitigate structural barriers. Collectively, such interventions may enhance academic integrity, promote more equitable research careers, and better align authorship practices in Germany with international standards.

## Supplementary Information

Additional file 1: Questionnaire.

Additional file 2: STROBE_checklist_cross-sectional.

## Data Availability

The datasets generated and analyzed during the current study are available from the corresponding author on reasonable request.

## Abbreviation

NRW: North Rhine-Westphalia

## References

[1] Chapman CA, Bicca-Marques JC, Calvignac-Spencer S, Fan P, Fashing PJ, Gogarten J, et al. Games academics play and their consequences: how authorship, h-index and journal impact factors are shaping the future of academia. Proc Biol Sci. 1916;2019(286):20192047. 10.1098/rspb.2019.2047 PMC6939250 31797732

[2] Aliukonis V, Poškutė M, Gefenas E. Perish or publish dilemma: challenges to responsible authorship. Medicina (Kaunas). 2020. 10.3390/medicina56030123. 10.3390/medicina56030123 PMC7142498 32178434

[3] Brunson JC, Wang X, Laubenbacher RC. Effects of research complexity and competition on the incidence and growth of coauthorship in biomedicine. PLoS ONE. 2017. 10.1371/journal.pone.0173444. 10.1371/journal.pone.0173444 PMC5362051 28329003

[4] Greenblatt DJ. Authorship. Clin Pharmacol Drug Dev. 2022;11(12):1362–6. 36448960 10.1002/cpdd.1190

[5] Papatheodorou SI, Trikalinos TA, Ioannidis JPA. Inflated numbers of authors over time have not been just due to increasing research complexity. J Clin Epidemiol. 2008;61(6):546–51. 18471658 10.1016/j.jclinepi.2007.07.017

[6] Salas SP. Ethics of authorship. In: Valdés E, Lecaros JA, editors. Handbook of Bioethical Decisions Volume II: Scientific Integrity and Institutional Ethics. Collaborative Bioethics. Cham: Springer International Publishing; 2023:37–47.

[7] Bössel N, Kluge A, Leising D, Mischkowski D, Phan LV, Schmitt M, et al. Anreizsystem, Machtmissbrauch und wissenschaftliches Fehlverhalten Eine Analyse zum funktionalen Zusammenhang zwischen strukturellen Bedingungen und unethischem Verhalten in der Wissenschaft. DGPs-Kommission. 2022.

[8] Kwok LS. The White Bull effect: abusive coauthorship and publication parasitism. J Med Ethics. 2005;31(9):554. 16131560 10.1136/jme.2004.010553 PMC1734216

[9] Drenth JP. Multiple authorship: the contribution of senior authors. JAMA. 1998;280(3):219–21. 9676660 10.1001/jama.280.3.219

[10] Baerlocher MO, Newton M, Gautam T, Tomlinson G, Detsky AS. The meaning of author order in medical research. J Investig Med. 2007;55(4):174–80. 10.2310/6650.2007.06044 17651671

[11] Bhattacharya S. Authorship issue explained. Indian J Plast Surg. 2010;43(2):233–4. 21217997 10.4103/0970-0358.73482 PMC3010799

[12] International Committee of Medical Journal Editors. Recommendations for the Conduct, Reporting, Editing, and Publication of Scholarly Work in Medical Journals. Updated January 2024. https://www.icmje.org/recommendations/. Accessed 13 May 2026. 10.12771/emj.2024.e48 PMC12093628 40704003

[13] Bhopal R, Rankin J, McColl E, Thomas L, Kaner E, Stacy R, et al. The vexed question of authorship: views of researchers in a British medical faculty. BMJ. 1997;314(7086):1009–12. 9112845 PMC2126416

[14] Mayyas F, Alzoubi K. Awareness and knowledge of manuscript writing and research integrity: a cross sectional survey among graduate students. Heliyon. 2022;8(11):e11447. 36406701 10.1016/j.heliyon.2022.e11447 PMC9667251

[15] Mubeen SM, Ghayas R, Adil Rizvi SH, Khan SA. Knowledge of scientific misconduct in publication among medical students. Educ Health (Abingdon). 2017;30(2):140–5. 28928344 10.4103/efh.EfH_221_16

[16] Helgesson G, Juth N, Schneider J, Lovtrup M, Lynoe N. Misuse of coauthorship in medical theses in Sweden. J Empir Res Hum Res Ethics Int J. 2018;13(4):402–11. 10.1177/1556264618784206 29985088

[17] Helgesson G, Holm S, Bredahl L, Hofmann B, Juth N. Misuse of co-authorship in medical PhD theses in Scandinavia: a questionnaire survey. J Acad Ethics. 2023;21(3):393–406.

[18] Yamamura J, Molwitz I, Ozga AK, Nguyen TA, Wedekind I, Wolf-Baldauf L, et al. Gender differences and cooperation in medical authorships - an analysis of the recent ten years in five key medical disciplines. BMC Med Educ. 2023;23(1):68. 36707803 10.1186/s12909-023-04041-6 PMC9883917

[19] Misra V, Safi F, Brewerton KA, Wu W, Mason R, Chan AW, et al. Gender disparity between authors in leading medical journals during the COVID-19 pandemic: a cross-sectional review. BMJ Open. 2021;11(7):e051224. 10.1136/bmjopen-2021-051224 PMC8282422 34261692

[20] Feldon DF, Peugh J, Maher MA, Roksa J, Tofel-Grehl C. Time-to-credit gender inequities of first-year PhD students in the biological sciences. CBE Life Sci Educ. 2017. 10.1187/cbe.16-08-0237. 10.1187/cbe.16-08-0237 PMC5332047 28130271

[21] Pezzoni M, Mairesse J, Stephan P, Lane J. Gender and the publication output of graduate students: a case study. PLoS ONE. 2016;11(1):e0145146. 26760776 10.1371/journal.pone.0145146 PMC4711938

[22] Hart KL, Boitano LT, Tanious A, Conrad MF, Eagleton MJ, Lillemoe KD, et al. Trends in female authorship in high impact surgical journals between 2008 and 2018. Ann Surg. 2022;275(1):e115–23. 32590539 10.1097/SLA.0000000000004057

[23] Hochschul-Bildungs-Report. Studierende: Verteilung insgesamt nach Fächergruppe 2025 19.03.2026. Available from: https://www.hsi-monitor.de/themen/internationale-studierende/studierende-grunddaten/verteilung-studierende-nach-fach/.

[24] Statistisches-Bundesamt. 4% mehr Promovierende im Jahr 2024. Pressemitteilung Nr298 vom 14 August 2025. 2025 23.03.2025. Available from: https://www.destatis.de/DE/Presse/Pressemitteilungen/2025/08/PD25_298_213.html.

[25] Leiner DJ. *SoSci survey* Computer software (Version 3.5.02). 2024.

[26] Chander S, Luhana S, Sadarat F, Leys L, Parkash O, Kumari R. Gender and racial differences in first and senior authorship of high-impact critical care randomized controlled trial studies from 2000 to 2022. Ann Intensive Care. 2023;13(1):56. 37368060 10.1186/s13613-023-01157-2 PMC10299980

[27] Kiermer V, Adams S, Bibbins-Domingo K, Flores Bueso Y, Jamieson KH, Heber J, et al. Creating a responsible authorship culture in science: anchoring authorship practices in principles of transparency, credit, and accountability. Proc Natl Acad Sci U S A. 2026;123(12):e2531268123. 41811430 10.1073/pnas.2531268123 PMC13012122

