# Correction: Who deserves credit, who receives credit? A cross-sectional survey on the handling of co-authorship in medical dissertations in Germany

**DOI:** 10.1186/s41073-026-00237-8

**Published:** 2026-07-27

**Authors:** Laura Klempp, Nadin Zerin Tanriverdi, Adrian Loerbroks, Niklas Juth, Nils Hansson

**Affiliations:** 1 Department of the History, Philosophy and Ethics of Medicine, Centre for Health and Society, Medical Faculty, Heinrich-Heine-University, Moorenstraße 5, Duesseldorf, 40225 Germany; 2 Institute of Occupational, Social and Environmental Medicine, Centre for Health and Society, Medical Faculty, Heinrich-Heine-University Duesseldorf, Duesseldorf, Germany; 3 Department of Public Health and Caring Sciences, Centre for Research Ethics and Bioethics, Uppsala University, Uppsala, Sweden

**Correction: Res Integr Peer Rev 11, 34 (2026)**

**https://doi.org/10.1186/s41073-026-00219-w**

Following publication of the original article [1], it was reported that an incorrect version of Fig. 1 was used.

The incorrect Fig. 1 was:
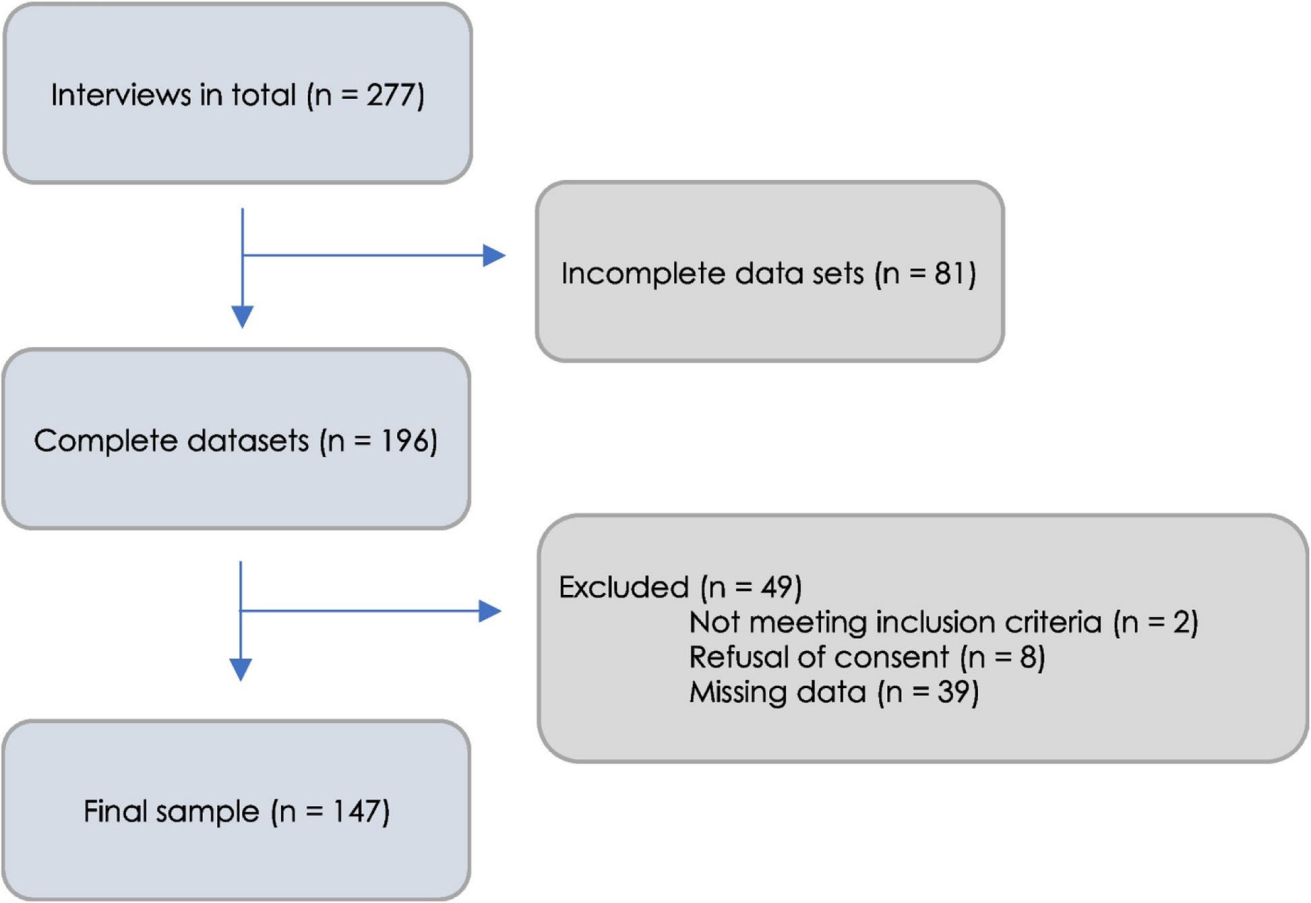


The correct Fig. 1 is:
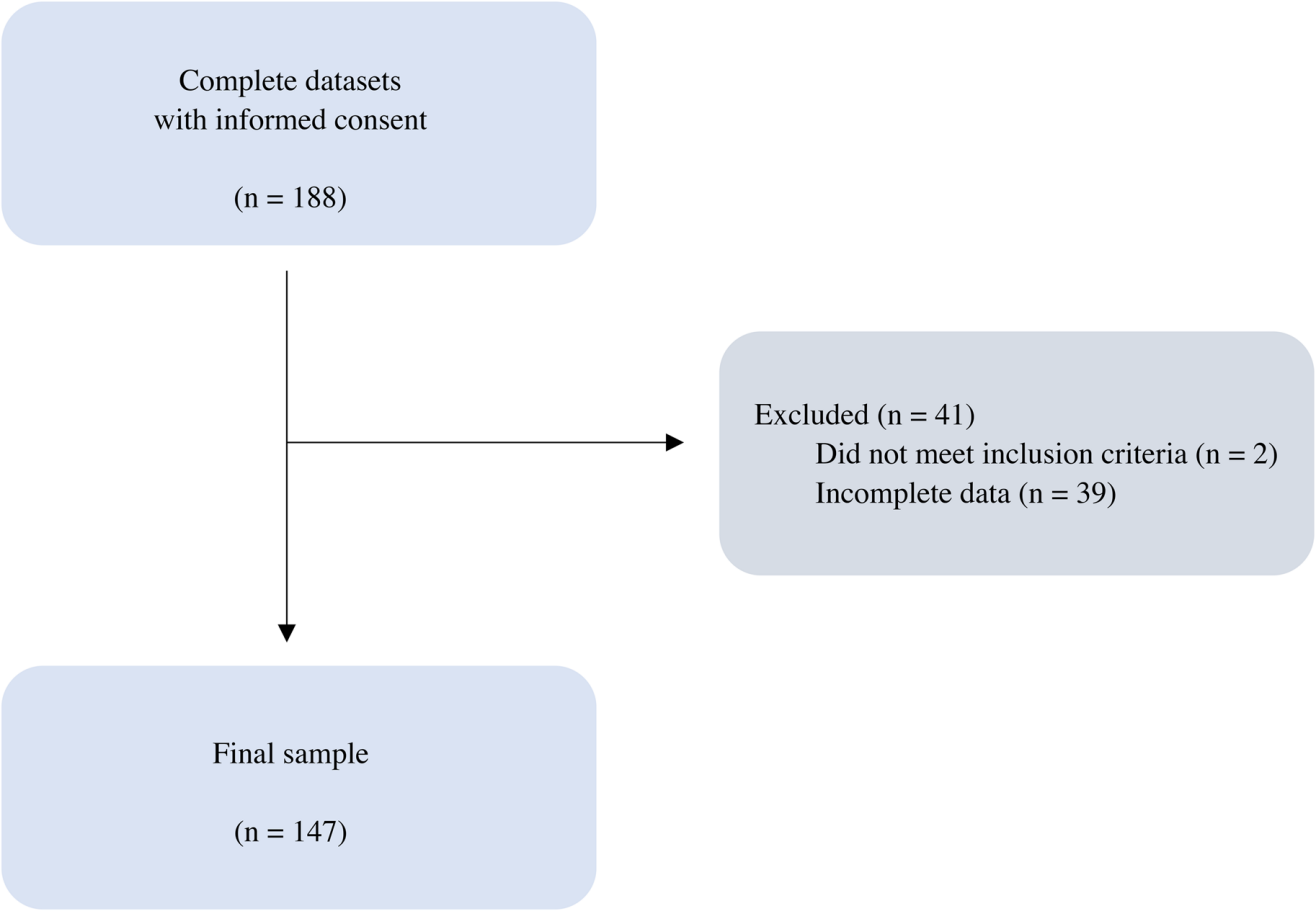


The original article has been updated.
