# Supplementary material for: Who deserves credit, who receives credit? A cross-sectional survey on the handling of co-authorship in medical dissertations in Germany
Source: Res Integr Peer Rev. 2026 May 22;11:34. doi: 10.1186/s41073-026-00219-w (PMC13195902; doi:10.1186/s41073-026-00219-w)
Supplement: Supplementary file 1 — Additional file 1: Questionnaire. [file 41073_2026_219_MOESM1_ESM.docx]

Questionnaire:

Welcome to the survey for the study on “Misuse of Co-Authorship in Medical Doctoral Theses”. Completing the questionnaire will take approximately 10 minutes.

Please answer to the best of your knowledge and based on your opinion.

All responses will be treated anonymously, including any open-ended comments. if you still choose to leave text comments, please make sure not to include any information that could be used to personally identify someone.

Thank you for your help.

1. Gender
   1. Female
   2. Male
   3. Divers
   4. Prefer not to say
2. Has a scientific publication in a peer-reviewed journal or a monograph resulted from your dissertation?
   1. Yes
   2. No
3. What was your position in the author list?
   1. First author
   2. Last author
   3. Corresponding author
   4. Other
4. What type of doctorate did you complete?
   1. Dr. med.
   2. Dr. med. dent
   3. PhD
   4. Dr. PH
5. Where did you defend your doctoral thesis? (Institution where you were enrolled as a doctoral candidate)
   1. RWTH Aachen
   2. Ruhr University Bochum
   3. University of Bonn
   4. Heinrich Heine University Duesseldorf
   5. University of Cologne
   6. University of Muenster
   7. University of Witten/Herdecke
   8. University of Bielefeld
   9. Outside North Rhine-Westphalia
6. In which year did you defend your doctoral thesis?
   1. 2024
   2. 2025
7. Gender of your primary supervisor?
   1. Female
   2. Male
   3. Diverse
   4. Prefer not to say
8. How would you describe your dissertation?
   1. Basic research
   2. Clinical research
   3. Other applied research
   4. Mixed (basic and clinical/applied research)
   5. Other

In the next section, following information about the so-called Vancouver Guidelines, questions regarding the handling of co-authorships in medical research publications will be asked. After each section, there will be the opportunity to leave open comments.

According to the Vancouver Guidelines issued by the International Committee of Meical Journal Editors (ICMJE), the following four criteria must all be fulfilled for someone to qualify as a co-author in biomedical journals:

1. Substantial contributions to the conception or design of the work; or the acquisition, analysis, or interpretation of data for the work;
   AND
2. Drafting the work or revising it critically for important intellectual content;
   AND
3. Final approval of the version to be published;
   AND
4. Agreement to be accountable for all aspects of the work, ensuring that questions related to the accuracy or integrity of any part of the work are appropriately investigated and resolved.
5. As a doctoral candidate, did your ever receive information about the Vancouver Guidelines or other ethical guidelines for co-authorship?
   1. Yes
   2. No
6. Were clear co-authorship guidelines/strategies applied in your department?
   1. Yes, consistently
   2. Yes, partially
   3. No
   4. I don’t know / Not sure
7. In your opinion, how reasonable are the Vancouver Guidelines?
   1. Very reasonable
   2. Quite reasonable
   3. Somewhat reasonable
   4. Not very reasonable
   5. Not reasonable at all

You indicated that you don not find the Vancouver Guidelines reasonable. What are your main reasons? (Open comment)

1. In your opinion, was anyone listed as a co-author on any of the papers included in your dissertation:
   1. Without having made a substantial contribution to the conception/design of the work or the acquisition, analysis, or interpretation of the data?
      - Yes
      - No
      - I don’t know / Not sure
   2. Without drafting the work or revising it critically for important intellectual content?
      - Yes
      - No
      - I don’t know / Not sure
   3. Without giving final approval of the version to be published?
      - Yes
      - No
      - I don’t know / Not sure

Comments: (Please ensure no identifying information about individuals or institutions is included, to maintain anonymity.)

1. In your opinion, how important is it that co-authorship is handled according to the Vancouver Guidelines?
   1. Very important
   2. Quite important
   3. Somewhat important
   4. Not very important
   5. Not important at all

In medical research, there is broad agreement that the order of authors listed on a scientific publication should reflect the relative contribution of each author to the research presented (including drafting the paper itself).

1. In your opinion, did any of the papers in your dissertation list authors in an order that did not reflect their actual contributions?
   1. Yes
   2. No
   3. I don’t know / Not sure
2. In your opinion, how important is it that the order of authorship reflects each authors relative contribution?
   1. Very important
   2. Quite important
   3. Not very important
   4. Not important at all

This was the final question.

If you have any questions about the survey, feel free to contact us.

Thank you very much for your help.
